# Development of a novel primate welfare assessment tool for research macaques

**DOI:** 10.1017/awf.2024.3

**Published:** 2024-01-24

**Authors:** Emilie A Paterson, Carly I O’Malley, Dawn M Abney, William J Archibald, Patricia V Turner

**Affiliations:** 1 Dept of Pathobiology, University of Guelph, Guelph, ON, N1G 2W1, Canada; 2 Global Animal Welfare & Training, Charles River, Wilmington, MA, 01887, USA; 3 Charles River, Reno, NV, 89511, USA; 4 Charles River, Tranent, East Lothian, EH33 2QL, UK

**Keywords:** 3Rs, animal behaviour, animal welfare, behaviour management programmes, refinement, welfare assessment

## Abstract

Primates are important species for biomedical research and ensuring their good welfare is critical for research translatability and ethical responsibility. Systematic animal welfare assessments can support continuous programme improvements and build institutional awareness of areas requiring more attention. A multi-facility, collaborative project aimed to develop and implement a novel primate welfare assessment tool (PWAT) for use with research macaques. PWAT development involved: establishing an internal focus group of primate subject matter experts, identifying animal welfare categories and descriptors based on literature review, developing a preliminary tool, beta-testing the tool to ensure practicality and final consensus on descriptors, finalising the tool in a database with semi-automated data analysis, and delivering the tool to 13 sites across four countries. The tool uses input- and outcome-based measures from six categories: physical, behavioural, training, environmental, procedural, and culture of care. The final tool has 133 descriptors weighted based upon welfare impact, and is split into three forms for ease of use (room level, site level, and personnel interviews). The PWAT was trialled across facilities in March and September 2022 for benchmarking current macaque behavioural management programmes. The tool successfully distinguished strengths and challenges at the facility level and across sites. Following this benchmarking, the tool is being applied semi-annually to assess and monitor progress in behavioural management programmes. The development process of the PWAT demonstrates that evidence-based assessment tools can be developed through collaboration and consensus building, which are important for uptake and applicability, and ultimately for promoting global improvements in research macaque welfare.

## Introduction

Across contract research organisation (CRO) facilities it can be difficult to have uniform harmonisation of animal facility management and associated Standard Operating Procedures (SOPs) due to differences in client needs and study types, regional or national oversight body requirements, availability of resources for facility design and equipment, and differences in cultural practices across sites (Underwood 2007). Some harmonisation of animal care practices can be accomplished through local oversight bodies, such as animal ethics committees or through international third party animal welfare assessment organisations, such as AAALAC, International (Kendall *et al.* 2018; Bayne & Turner 2019; Mohan & Huneke 2019). However, these committees and organisations largely provide guidance and do not go into detail concerning welfare assessment or behavioural management programmes (Turner & Bayne 2023). This can result in a wide range of behavioural management practices and programmes and asynchronous improvements, which may contribute to variability in the scientific data outcomes as well as animal welfare (Everitt & Berridge 2017).

Approximately 100,000 primates are used in biomedical research around the world every year not including primates kept for breeding purposes (Lankau *et al.* 2014). The most common research primate species are cynomolgus macaques (*Macaca fascicularis*), rhesus macaques (*Macaca mulatta*), and common marmosets (*Callithrix jacchus)* (Lankau *et al.* 2014; Paterson & Turner 2022). Research primates across facilities may live in different housing conditions and undergo various study- and veterinary-related procedures (Carlsson *et al.* 2004; Johnsen *et al.* 2012; Wolf & White 2012). It is important to have accurate and ongoing assessments of the individual and group animal welfare states in these different circumstances to fulfil moral, legal, and scientific obligations (Turner 2020; Canadian Council on Animal Care [CCAC] 2021).

When assessing animal welfare, measures should be inspired by the framework of “a life worth living”, which incorporates positive welfare indicators (Mellor 2016; Nunamaker *et al.* 2021). Primates have complex requirements to meet their behavioural management needs, such as appropriate biological health and functioning, positive affective states, and the ability to display natural behaviours (Fraser *et al.* 1997; Howell & Cheyne 2019; Testard *et al.* 2021). Under natural conditions, many primate species, such as macaques and marmosets, live in small- to medium-sized social groups in which there is an established hierarchy and behavioural synchronisation (Lehmann *et al.* 2007). These interactions can impact access to resources, such as food (Hambali *et al.* 2012), and complex social behaviours, such as huddling during rest that can affect overall energy expenditure for thermoregulation (Campbell *et al.* 2018). Human-primate interactions can also impact welfare. Implementing behaviour-focused practices, such as positive reinforcement training (PRT) contributes to improved affective state (Prescott & Buchanan-Smith 2003; Perlman *et al.* 2012; Turner & Bayne 2023). PRT is a form of positive human-animal interaction that allows animals some choice and control, and its implementation can reduce overall daily stress related to husbandry and study procedures (Schapiro *et al.* 2005). In a study looking at PRT in chimpanzees (*Pan troglodytes*), animals who voluntarily presented for an anaesthetic injection had significantly lower white blood cell counts, absolute segmented neutrophil counts, and glucose levels indicative of less stress compared to the chimpanzees anaesthetised using traditional methods (Lambeth *et al.* 2006).

Welfare assessments are intended as a holistic evaluation of the impact of animal behaviour management and care programmes and underpin a continuous quality improvement model (i.e. plan-do-check-act). The assessments are different from daily observations that may be conducted as part of animal husbandry and humane intervention point assessments (CCAC 2021; Nunamaker *et al.* 2021) and ideally are formalised and documented, with definition of action plans. Animal welfare assessment should consider input- and outcome-based measures (Barnett & Hemsworth 2009; CCAC 2019, 2021). Input-based measures focus on the resources that animals are provided (Vasseur *et al.* 2012). These measures are reliable and have good inter- and intra-rater reliability; however, they can lack validity. Input-based measures are an indirect reflection of animal welfare as individual animals will perceive the environment differently, leading to a spectrum of welfare states (Vasseur *et al.* 2012; Prescott *et al.* 2022). For example, providing the adequate resources in the environment to encourage species-typical behaviour. Outcome-based measures are a direct reflection of one’s state (Vasseur *et al.* 2012; Prescott *et al.* 2022). For example, quantifying the use of the provided resources through behavioural observations. Another important outcome-based measure at the human level in a laboratory setting is the culture of care (Klein & Bayne 2007; Robinson *et al.* 2019; Bayne & Turner 2019). Having a good culture of care means that the institutional team (e.g. caregivers and technical personnel, researchers, animal ethics committee members, veterinarians, managers, etc) understand the importance of their work, and they are working together towards a common goal of promoting high-quality science and proactive animal care based on science-based performance standards that exceed basic regulatory requirements, and that the employees working directly with the animals are satisfied in their job in items such as comprehensive training to be able to care for animals well, work-life balance to avoid stress and rushing, resources related to compassion fatigue and resiliency building to promote good mental health for employees, and that employees feel valued and heard in their role of promoting good animal welfare (Bayne & Turner 2019; Robinson *et al.* 2019).

Welfare assessment tools have been developed for specific species such as elephants (Yon *et al.* 2019), horses (Long *et al.* 2022), reptiles (Benn *et al.* 2019), for animals housed in zoos (Sherwen *et al.* 2018), for livestock species in different stages of production (see, for example, Kirchner *et al.* 2014; Buijs *et al.* 2017; Kang *et al.* 2022), but only recently has attention turned towards primates in biomedical research settings (Truelove *et al.* 2020; Prescott *et al.* 2022). The main purpose of an animal welfare assessment tool is to have an objective and quantitative measure of animal welfare that permits regular assessment with the goal of taking action to improve animal welfare (Honess & Wolfensohn 2010; CCAC 2021).

The goal of this project was to develop a novel primate welfare assessment tool that could be applied across sites and countries to assess research primate (predominantly macaque) welfare at the facility level, and globally, refine primate welfare through knowledge of ‘current state’ and creating a culture of continuous improvement. The aim of implementing the tool is to provide a means for formalising primate welfare assessments and to harmonise considerations and approaches to primate care and welfare across sites and countries, regardless of intended research primate use.

## Materials and methods

### Focus group development

In May 2020, members of Charles River’s animal welfare oversight group, Global Animal Welfare and Training (GAW&T), hosted a two-day global internal and virtual 3Rs primate workshop. A significant deliverable of this workshop was to develop a Primate Welfare Assessment Tool (PWAT). Internal primate experts (i.e. primate behaviourists, primate behaviour champions, and veterinarians) were invited to participate on the project. Ten individuals were recruited to form a focus group from across sites in Canada (n = 4), Europe (n = 2), and the United States (n = 4). Two of the participants were the focus group co-leaders (EAP, PVT) with > 6 and > 28 years experience, respectively, of working with macaques in various research settings. After agreeing upon a charter, the focus group met virtually every three weeks for approximately eight months.

### Preliminary tool development

The preliminary tool was based on an extensive list of animal welfare descriptors to be used in a zoo setting as well as other settings, such as pigs on-farm (Association of Zoos and Aquaria [https://www.aza.org/accred-materials]; Kagan *et al.* 2015; Courboulay *et al.* 2020). The two main researchers (EAP, PVT) refined the list of descriptors based on the current literature and experience with macaques in a range of research facilities, including primate quarantine and procurement, discovery or exploratory settings, and safety assessment. The refined list of descriptors were categorised into physical, behavioural, environmental, training, procedural, and culture of care, which were further divided into subcategories (categories, category aims, subcategories, and maximum score that could be achieved per subcategory are provided in Table 1. For the full list of descriptors by category and subcategory, see Table 2. Every descriptor was attributed a weighted score from 1 to 5 based on the degree of impact on primate welfare based on the literature, with 1 being a very low impact on welfare and 5 very high (welfare weights per descriptor are provided in Table 2). A numerical scale was created for scoring, which included scores of 0, 1, and 2. A score of 0 was representative of something or an activity that was rarely present (< 25%), 1 was indicative of something moderately present (25–75%) and a score of 2 was representative of an item or activity that was obviously present (> 75%). A nominal scale was created for descriptors for which ‘yes’ (2) or ‘no’ (0) responses were applicable. A ‘non-applicable’ option was also available for certain descriptors and, if selected, the points from that descriptor were not added to the final score. Non-applicable was only used if the descriptor was not physically possible (e.g. maternal-offspring rearing opportunities at a facility in which no animal breeding occurred) or could not be evaluated during the assessment (e.g. assessment of animal behaviour during procedure when no procedures were occurring). Some descriptors were provided as bonus points if they were harder to achieve but were important for animal welfare (e.g. access to outdoor spaces, use of remote animal monitoring systems). One bonus descriptor subtracted points from the final score based on the number of singly housed animals within a room (maximum score reduction was set at 20 points). The tool was first created in Microsoft Excel® (Microsoft® Corporation, Redmond, Washington, USA, version 2019). The preliminary tool was presented to the focus group and consensus was achieved on the descriptors, how descriptors were best categorised, the welfare weight of each descriptor, and the numerical/nominal scale for each descriptor. The group suggested applying the tool every six months – a time-frame that would allow sites to select and make progress on several findings while not so long that the tool and its purpose could be forgotten.

**Table 1.** Aim and description of each category and subcategory included in the Primate Welfare Assessment Tool, as well as the maximum score, calculated by multiplying the weight of the descriptor with the highest score possible for that indicator

Category	Subcategories	Maximum Score
**Physical:** assess the physical or clinical health of the animals and factors that may influence health	General condition	44
	Nutrition	20
	Pain assessment and mitigation	20
	Records	16
**Behavioural:** assess normal and abnormal primate behaviour and potential behavioural management inputs that are reflected in the animals’ behaviour	Behavioural assessments	20
	Animal behaviour	16
	Social behaviour	32
	Feeding behaviour	6
	Parental behaviour	12
	Ability to cope	8
	Bonus	+5
**Environmental:** assess the quality of the space provided and the required components and resources that allow them to express species-typical behaviour	Housing	82
	Resources/enrichment	96
	Exercise opportunities	16
	Bonus	−20 +38
**Training:** assess various aspects of the training procedures in place to decrease stress and other negative affective states as well as giving primates control over their environment	Acclimation	12
	Habituation, desensitization, and counter conditioning	56
	Positive reinforcement training	28
	Human interactions	18
	Animal participation and co-operation	22
**Procedural:** assess the procedures (or techniques) used at the facility to ensure they are in line with recent literature	Capture	8
	Restraint	18
	Procedures	44
	Recovery	8
	Ambience	14
	Scheduling	12
**Culture of care:** assess the training and resources provided to the employees to allow them to feel job satisfaction and confidence in their work with primates and to promote compassion satisfaction and resilience	Initial training	46
	Continuing education	12
	Compassion fatigue and resiliency building programmes/activities	32
	Involvement/opportunity	16
	Choice and control in work schedule	30
	Recognition	16
	Voice concerns	24
	Competencies	14
	Bonus	+28

**Table 2.** Full list of Primate Welfare Assessment Tool descriptors, including welfare weight (1–5, with 1 being low impact on welfare and 5 being high impact on welfare) and assessment form (room, site, or culture of care personnel interviews). The table is divided by category: 2(a) Physical table, 2(b) Behaviour, 2(c) Environmental, 2(d) Training, 2(e) Procedural and 2(f) Culture of care

Table 2(a)		
Category PHYSICAL	Weight	Form
**Subcategory**: General condition Primates are fully upright (not hunched over and heads are not tucked into limbs or into corner of cage	4	Room
Primates have a clean and largely intact hair coat	2	Room
Primates readily move towards or away from observer (depending on temperament), react normally to external stimulus	3	Room
Primates have appropriate muscular development and fat deposit for sex and age.	3	Room
Primates do not appear to be dehydrated (i.e. eyes are forward and do not appear sunken)	3	Room
No signs of gastrointestinal, skin, neurologic, urogenital, musculoskeletal or respiratory conditions	3	Room
Primates are bright, alert, and responsive to observer	4	Room
**Subcategory** Nutrition The animals receive different types of food (fruits, vegetables, rewards) that provides variety and novelty for them	3	Site
Lack of excessive base diet untouched in enclosure	4	Room
Food evaluations are conducted regularly, especially after a procedure that could cause reduced appetite. An established procedure is in place for primates with reduced appetite (i.e. supplementation)	3	Site
**Subcategory** Pain assessment and mitigation Care personnel are competent in recognising pain	3	Site
The facility has a specific policy or SOP concerning primate pain management practices based on current veterinary practices	4	Site
Primates receive individualised doses of pain medications (i.e. per kg)	3	Site

Table 2(b)		
Category BEHAVIOUR	Weight	Form
**Subcategory Behavioural assessment** Personnel are specifically trained to identify normal and abnormal behaviours	4	Site
Behavioural assessments are conducted regularly for all animals and abnormal behaviours are documented	2	Site
There is a team or individual that specialises in primate behaviour	4	Site
**Subcategory Animal behaviour** Animals are not displaying abnormal/stereotypical behaviour, (i.e. locomotor stereotypy, self-mutilation, appetitive stereotypy, self-directed stereotypy, hair plucking, etc)	4	Room
Animals do not show maladaptive or abnormal behaviours during times of non-activity in the room (e.g. hair pulling, self-mutilation, self-directed stereotypy)	4	Room
**Subcategory Social behaivour.** Primates are housed with at least one other compatible social partner (displaying positive social behaviours, e.g. grooming, playing)	5	Room
Compatibility of social groupings is assessed regularly and there is an established procedure if social partners are incompatible	3	Site
There is no overt evidence of fighting, such as tail tip traumas or facial trauma	2	Room
There is no evidence that a specific primate in a group is underfed or bullied due to the social hierarchy	3	Room
Quality of life is assessed for low-ranking animals (e.g. have access to food, are not overtly bullied, willing to share space with dominant animals)	3	Room
**Subcategory Feeding behaviour** Primates are expressing natural feeding behaviours	3	Room
**Subcategory Parental behaviour** Primates have the opportunity to express parental behaviour	3	Site
Primates are reared within a natural time-span	3	Site
**Subcategory Ability to cope** When separated from social partners for a procedure, primates do not perform abnormal behaviours	4	Room
**Subcategory Bonus** The facility has an indirect monitoring system permitting indirect observations (limiting observer effect on primate behaviour)	5	Site

Table 2(c)		
Category ENVIRONMENTAL	Weight	Form
**Subcategory Housing** Animals are housed in pens	4	Site
The enclosure maximises vertical space use	5	Site
The enclosures have visual barriers and privacy areas for the animals to hide from other primates and humans and escape aggressors	5	Site
There are multiple feeding, drinking and resting areas in enclosures to maximise access to resources	3	Site
Animals can walk, run, climb, leap, swing, and hang in home enclosures	3	Site
When separated, animals still have visual, auditory, olfactory, and/or tactile contact with conspecifics	3	Site
Enclosures are a suitable size for the weight and/or age of the primates, enabling them to perform postural changes with ease	5	Site
The room that animals are housed in is not excessively noisy (i.e. crowds, construction, vocalisations from other species)	2	Site
When animals are singly housed, there is an SOP/procedure to find a companion and/or extra positive human interaction	5	Site
When animals are singly housed, there is an SOP/procedure for extra resources/enrichment	3	Site
Animals can avoid being disturbed by animal care activities (e.g. during room maintenance, animals can avoid being wetted)	3	Site
**Subcategory Resources/enrichment** There is a schedule that staff follow to ensure variety and stability in resources/enrichment provision	4	Site
The behavioural management and/or enrichment programme is regularly evaluated and reviewed	4	Site
The resources/enrichment provided are documented and reviewed	3	Site
The facility has an established structured resource/enrichment group	4	Site
Resources/enrichment provided are trialed and validated before widespread use	4	Site
Natural materials are provided (i.e. wooden structures)	3	Site
Thermoneutral surfaces (i.e. surfaces that do not alter animal body temperature) present to promote comfort	4	Site
Opportunities to forage are provided (i.e. large surface area where animals can search for food)	5	Site
Elevated benches/perches/resting boards are provided at appropriate heights	5	Site
A balcony or tunnel is present permitting a view into the room or outside	3	Site
Primates are provided with species and age-specific sensory resources (i.e. visual, auditory, etc)	2	Site
Cognitive stimulation is provided to the primates through positive reinforcement training or technology	3	Site
There is at least one manipulable item for each animal in the enclosure	4	Site
Manipulanda are regularly rotated to prevent boredom	3	Site
**Subcategory Exercise opportunities** Enclosure space exceeds regulatory requirements	4	Site
Animals have access to additional space outside of their home cage for exercise and all animals are provided with at least weekly access (applicable to only cage style housing not pens)	4	Site
**Subcategory Bonus** How many primates are singly housed in the room	−2	Room
Primates have access to natural breeding opportunities	5	Room
Primate rooms have windows or sources or natural light	5	Room
Primates are housed in mixed sex and age groups (greater than 5 animals per enclosure)	3	Room
Access to an outdoor enclosure is provided	25	Room

Table 2(d)		
Category TRAINING	Weight	Form
**Subcategory Acclimation** Site has an SOP/BOP outlining environmental acclimation plan	2	Site
Primates are given a minimum of 14 days of acclimation to the facilities after arrival to the site (i.e. vet care, husbandry procedures, positive human interaction only). This period is free from study activity	4	Site
**Subcategory Habituation, desensitisation, and counter-conditioning** Site has SOP/BOP specific to habituation and desensitisation of animals to study procedures (e.g. people, devices, procedures)	2	Site
Primates are habituated to any/all worn restraint devices (i.e. collars)	4	Site
Staff performing habituation activities are properly trained on purpose of habituation and how to correctly perform	4	Site
Habituations are performed in a controlled, quiet environment and are not paired with other activities (e.g. bodyweights, blood draws, collar placement)	2	Site
Staff document animal behaviour and progress during habituation	3	Site
Facility has a formal plan for steps to take for primates not habituating well or that require additional habituation sessions	3	Site
At completion of habituation to a procedure, primates are free from rectal prolapse and injury, and tolerate the procedure	3	Room
Some staff are specifically assigned to the same animal rooms to ensure familiarity of animals to people and people to animal needs	4	Site
Habituation and training of learned behaviours is maintained throughout animals’ time at facility	3	Site
**Subcutaneous Positive reinforcement training (PRT)** Staff using positive reinforcement training are trained on proper techniques	4	Site
Positive reinforcement training is used for animal housed long-term and/or for those in more challenging studies	3	Site
Animals are trained for behaviours that facilitate husbandry or veterinary procedures (e.g. shifting) using positive reinforcement training	3	Site
Training programmes are evaluated on a regular basis for adequacy	4	Site
**Subcutaneous Human interactions** Human interaction training is available and defines how to build trust and maintain positive interactions with animals at arrival and throughout time at the facility	4	Site
Primates readily come to enclosure front to accept treats and personnel documents animals not responding well to human interaction	2	Room
Primates are free from stress/fear related behaviour when staff are near enclosure	3	Room
**Subcutaneous Animal participation/co-operation** When removing primates from enclosures, animals cooperate with handling without signs of fear or distress (e.g. no vocalisations, no faeces present)	4	Room
Primates are calm when placed into a restraint device (i.e. chair)	4	Room
Primates are calm when undergoing procedures	3	Room

Table 2(e)		
Category PROCEDURAL	Weight	Form
**Subcategory Capture** Animals are comfortable and compliant with removal from home enclosure	4	Room
**Subcategory Restraint** The manual restraint provides full support to the animals’ head and the majority of bodyweight	4	Site
Restraint devices are comfortable (i.e. the animal has support standing & sitting in a natural position, soft surfaces, thermoregulated surfaces)	3	Site
If moderate restraint will occur, the restraint device has something to occupy animal engagement (e.g. attached manipulanda)	2	Site
**Subcategory Procedures** Staff are trained to respond appropriately to adverse situations (i.e. applying pressure during bleeding)	3	Room
For repeated blood collections, catheters are in use whenever possible. If not possible, there is a record of what vein was used and subsequent collections are performed on alternate veins to avoid repeated use of one vein	4	Site
During or following a procedure, animals are provided with a reward	3	Room
When removing food from animals for study activities, animals are paired (even if only one primate is undergoing the activity)	4	Site
When separation is needed for an activity/veterinary care, animals are separated for the least amount of time needed	4	Site
Animals are trained to take non-test article related substances voluntarily to reduce stress/manipulation (i.e. analgesics, antibiotics)	2	Site
Study activities are scheduled together as much as possible to minimise disturbance of animals	2	Site
**Subcategory Ambience** Following procedures, animals are monitored for pain indicators and to ensure animals return to normal state. Monitoring is performed by an individual familiar with the animals	4	Site
During procedures, noise levels are low	3	Site
There is sufficient space in the procedure areas for animals and humans to move easily without risk of injury	3	Site
Invasive procedures are conducted outside the main holding area	4	Site
**Subcategory Scheduling** The scheduling team and the vivarium team communicate to ensure that enough time is given to conduct procedure	2	Site
There are sufficient personnel scheduled to conduct study activities to avoid rushing	4	Site

Table 2(f)		
Category CULTURE OF CARE	Weight	Form
**Subcategory Initial training** Personnel are comfortable working with primates	4	CoC
Training includes species-specific behavioural training	5	CoC
Employees are allotted enough time to complete skills training	4	CoC
If employees do not feel comfortable after initial training, employees can have additional training or other options to accommodate concerns	3	CoC
Training is conducted in an environment conductive to learning (e.g. not rushed, competitive with other trainees, chaotic)	3	CoC
Employees are evaluated on competency (animal welfare, animal care, animal behaviour, procedures) following training(s) and if competency is poor, aspects of the training are repeated	4	CoC
**Subcategory Continuing education** Time is provided for personnel to complete continuing education (CE) during normal working hours	3	CoC
Primate-specific resources and CE material are readily accessible for employees	3	CoC
**Subcategory Compassion fatigue/resilience building programme/activities** Employees feel that they have a good work life balance	5	CoC
There is a process in place for employees to step away from or ask for help with an activity when feeling overwhelmed	3	CoC
Personnel can identify their Resiliency Building Ambassadors	3	CoC
Site-specific events occur to honor the animals that employees work with daily	2	CoC
Employees have access to resources and CE on compassion fatigue and resiliency building strategies	2	CoC
**Subcategory Involvement/opportunity** Employees actively participate in resource (’enrichment’) evaluation and improvement (i.e. new manipulanda, cage furniture, or foods)	3	CoC
Employees are actively involved in procedure development and refinement	3	CoC
Technical staff are encouraged and given time to attend/participate at pre-study meeting and can provide input on the study plan	2	CoC
**Subcategory Choice and control in work/schedule** Employees do not feel rushed or solely task-oriented. They have time to spend with the animals to provide additional positive interactions	5	CoC
Employees are connected to how their work supports human and animal health	4	CoC
Employees can choose to specialise with a preferred species	3	CoC
Employees can choose to opt-out of performing activities that they are uncomfortable doing without repercussions (e.g. euthanasia)	3	CoC
**Subcategory Recognition** The site participates in the corporate PAWS award programme and/or in a site-specific award program that recognises employee excellence when working with animals	4	CoC
Employees feel valued for their work	4	CoC
**Subcategory Voice concerns** Employees are comfortable reporting animal welfare concerns without fear of reprisal	4	CoC
Technical personnel have an equal or valued role when concerns are raised	4	CoC
Employees feel their concerns are addressed	4	CoC
**Subcategory Competencies** Facility personnel participate in meetings with other sites to discuss challenges and improvements on various procedures (e.g. behavior groups, vet services, etc)	3	CoC
Techniques are constantly being assessed for improvement and refinement, in line with current and emerging 3Rs practices and literature	2	CoC
Technical staff have a platform to ask questions, comment or share information regarding procedures	1	CoC
Technical staff can readily access SOP/BOP during their day-to-day work	1	CoC
**Subcategory Bonus** Site offers access to physical activities at times in which all staff can attend (i.e. access to fitness facilities/paid gym membership, on-site yoga classes)	7	CoC
Supervisors have attended the Frontline Leaders Workshop on creating an emotionally engaged culture through empathetic listening and the CR C.A.R.E.S. programme	7	CoC

### Beta-testing

The preliminary tool was beta-tested at six different facilities (two sites in Canada, one in UK, one in France, and one in the US) in December 2020 by six different participants. All facilities met or exceeded country-specific animal regulations and legislation and all were accredited by AAALAC International. Participants at small facilities reported that the assessment took approximately 2 h to complete while participants at larger facilities reported that it took 5–8 h. The most important and universal feedback from participants during beta-testing was that input- and outcome-based measures were hard to obtain when combined in the categories and that users wanted an easier platform for the assessment that minimised lateral scrolling. To address this challenge, the tool was divided into three assessment forms including room-level, in which animal outcome-based measures are evaluated, site-level, in which records reviews and input-based measured are evaluated, and a culture of care assessment, in which facility personnel feedback was sought and evaluated via an anonymous survey. Materials were translated into French to allow for multi-language participation.

### Pilot launch of finalised tool and implementation

**Figure 1.**
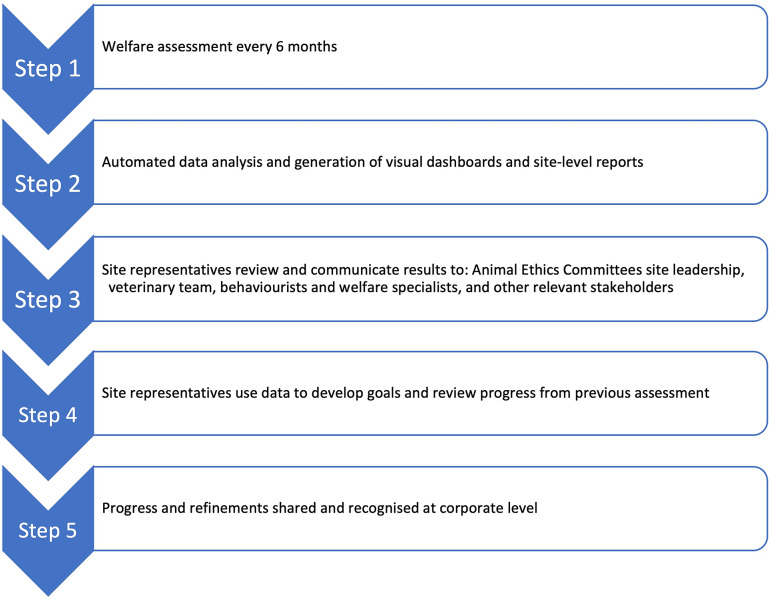
Primate welfare assessment process to be completed semi-annually to track welfare progress over time for facilities working with primates.

A finalised tool was created in Smartsheet® (Smartsheet Inc, Bellevue, WA, USA, version 2020), a practical platform allowing for easy data collection and automated tabulation and analysis of results. The answers from the three forms were collected into data-sheets with equations formatted to multiply answers by the designated welfare weight. The raw data were then referenced to metric sheets per category where the data were summed to give a total score for each category and subcategory. The summations were then referenced to dashboards where data were visualised as graphs for each facility and presented to senior global management for approval. The finalised tool in Smartsheet® was piloted at one facility in each of Canada and France. After focus group discussion and pilot-testing, several descriptors were eliminated due to redundancy or reworded for clarity. The final tool had 133 descriptors (Table 2). In March (Q1) and September (Q3) 2022, the PWAT was launched to 13 sites globally and each site was given 30 days to complete all forms. Sites were responsible for selecting whom would complete the assessment at their facility. Sites were asked to assess approximately10% of their occupied primate rooms (minimum of three rooms, maximum of ten depending on total number of occupied primate rooms) including a variety of housing types, animal purposes, study types, and study lengths, with one form submitted per room. Sites were also asked to submit one form for the site-level assessment, and to conduct 3–6 culture of care interviews depending on number of personnel working with primates (one form submitted per interview). The initial launch in 2022 served as benchmarking of current programmes to allow for monitoring over time for future assessments. This was done by averaging data across the two assessments (where two assessments were conducted in 2022). If a site only completed one of the two assessments, that one assessment was used as the overall 2022 benchmarking score for a year-end total result and report. Facilities were also asked to review the results with relevant stakeholders at their facility and identify up to three goals for enhancing their primate behavioural management programme in 2023 based on their specific results from the benchmarking assessments. The welfare assessment process is outlined in Figure 1.

### Tool-use training

English and French version user training guides were created that included a knowledge check (i.e. quiz with five questions) upon completion. The training was presented as an e-learning module and took approximately 10–15 min to complete. Each site designated 1–3 primary behaviour contacts for completing the tool and retained the same primary contacts in Q1 and Q3; however, some sites with three assessors added or changed their third assessor in Q3. The training guide included the learning outcomes, an explanation of the purpose of the PWAT, description of what was included in the tool, expectations and instructions for using the PWAT including the number of rooms, different types of studies, and employees to interview based on the facility size and function. Also included was a demonstration of how to access the results, and information on what to do with the results once received, such as communicating results to relevant stakeholders and developing discussions on next steps and top priorities to address, and finally a five question knowledge check. Participants needed to achieve 100% on this quiz to gain access to the tool.

## Results

Ten sites fully completed both assessments in 2022, although all 13 sites fully completed at least one of the two assessments. Misunderstandings regarding when completion was due or whom was completing the assessment contributed to the missing assessments for three facilities. For each assessment period, across all 13 facilities, an averaged total of 2,615 primates (all cynomolgus macaques) were assessed from within 62 rooms, and an averaged total of 66 employees completed the culture of care surveys.

**Figure 2.**
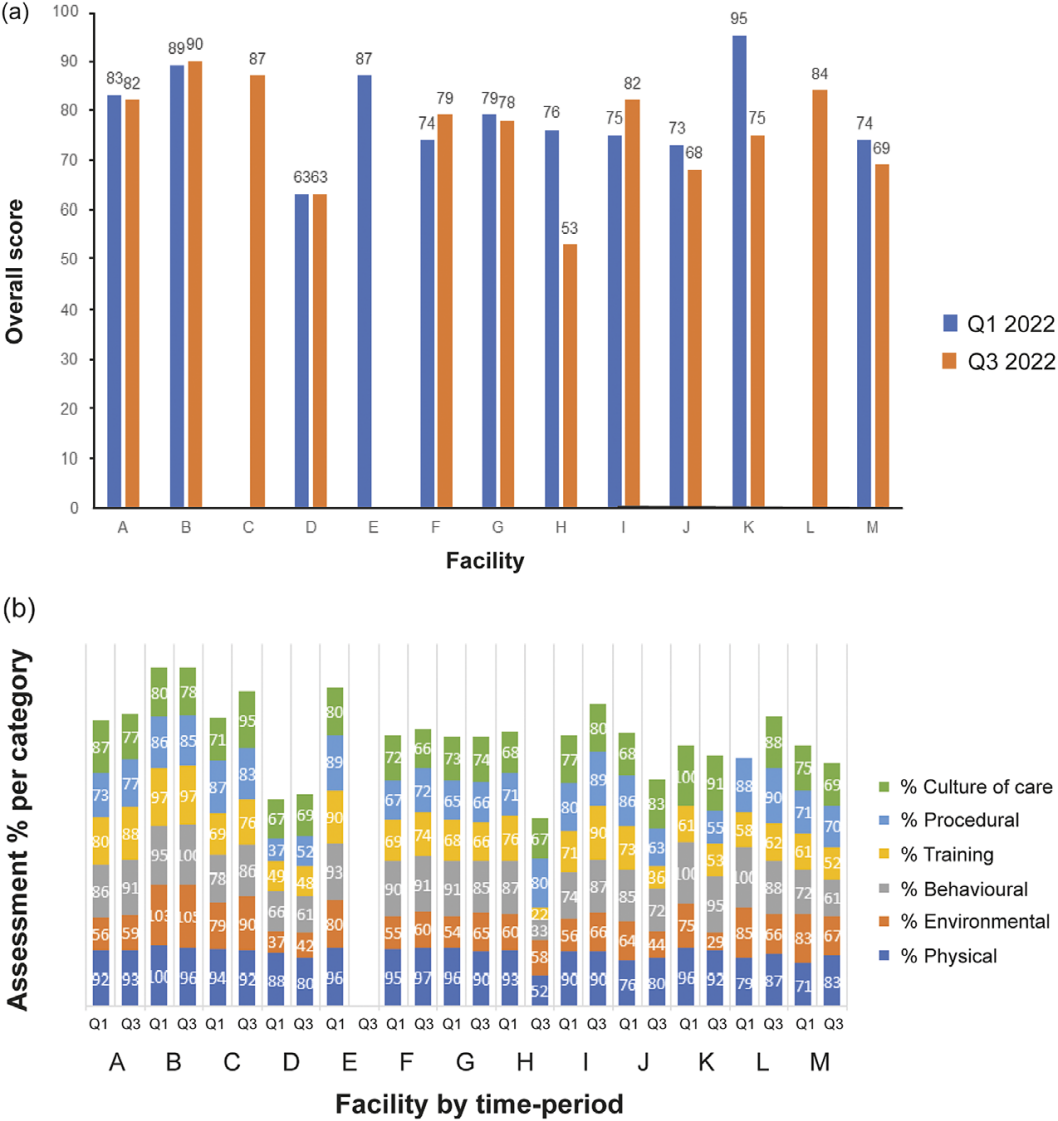
Total PWAT scores by site for the 2022 benchmarking for March (Q1 2022) and September (Q3 2022) (n = 13) including (a) the overall scores and (b) the relative scores by category (total possible score is 600).

The overall scores for Q1 and Q3 2022 are provided in Figure 2(a) and for each category in Figure 2(b). The breakdown of scores by category and subcategory for each facility for the combined 2022 benchmarking score are provided in Table 3. For Q1 2022, the scores ranged from 63–95%. For Q3 2022, the scores ranged from 53–90%. When looking at the categories assessed, sites performed best in the physical category (88.5% averaged across sites) and had the most room for improvement in the environmental category (65.2% averaged across sites). Two sites were outliers overestimating their welfare score (89 and 95%) due to misunderstandings in how to use the non-applicable score options. A follow-up meeting with PWAT site representatives was conducted to provide additional training. A summary report for each facility was provided for each assessment including the averaged 2022 results for overall PWAT score, welfare categories, and welfare subcategories. General findings are discussed below per category.

**Table 3.** Results of the 2022 welfare assessment, presented as the averaged percentage scores from the Q1 2022 and Q3 2022 assessments for each category, subcategory, and total score, presented for each facility (A–M) and averaged across facilities

Category	Subcategory	A	B	C	D	E	F	G	H	I	J	K	L	M	Avg
Physical	General condition	93.5	100	95	80	97	97.5	94.5	79.5	94	76	96.5	87	81.5	90.2
	Nutrition	92	100	100	95.5	92	91.5	89	60.5	94.5	72.5	100	100	44.5	87.1
	Pain assessment and mitigation	80	80	100	80	100	100	100	80	100	100	60	60	100	87.7
	Records	93.5	100	65	87	93	88	89.5	46.5	60	78	97	88	77	81.7
	Total	92.5	98	93	84	96	96	93	72.5	90	78	94	87	77	88.5
Behavioural	Behavioural assessments	70	100	60	35	100	80	90	55	70	90	100	100	100	80.8
	Animal behaviour	89	100	92	71.5	100	90.5	83	41.5	91.5	79	100	80	50	82.2
	Social behaviour	91.5	94.5	90	76.5	93	91.5	89	74.5	75	86.5	94	85	66	85.2
	Feeding behaviour	91.5	100	83	5	70	97.5	98	70	75	66.5	100	100	83.5	80
	Parental behaviour	na	na	na	na	na	na	100	na	na	na	na	na	na	na
	Ability to cope	85	100	83	50	80	82.5	72.5	33.5	91.5	46	100	100	50	74.9
	Bonus	0	0	0	5	0	0	5	5	0	0	0	0	0	
	Total	88.5	97.5	82	66	93	90.5	88	60	80.5	54.5	57	62	56.5	68.5
Environmental	Housing	62	100	91	44	90	69	71.5	76.5	76.5	62	69.5	95	81.5	76
	Resources/enrichment	64.5	92	80	57.5	77	73	70	56	59	56	61	64	79.5	68.4
	Exercise opportunities	25	100	na	0	50	25	25	12.5	62.5	12.5	25	50	37.5	35.4
	Bonus (+)	0	15	8	0	0	0	5	0	0	0	0	0	15	
	Bonus (-)	0	0	0	−20	0	−20	−20	0	−2	−6	−20	−20	−20	
	Total	57.5	104	84.5	37	80	57.5	59.5	59	61	54	52	66	75	65.2
Training	Acclimation	67	100	100	16.5	100	100	67	50	100	100	0	100	100	76.9
	Habituation, desensitisation, counter-conditioning	88	96	79	46.5	90	66.5	78	52	75.5	69	32	0	na	64.4
	Positive reinforcement training	57	89	64	15	0	31	52	27	0	50	50	0	50	37
	Human interaction	88	100	80	71.5	90	78	69.5	47	85.5	63	71.5	84	61	76.1
	Animal participation/co-operation	87	100	71	49	90	75	60	68	88.5	33	70	na	43.5	69.6
	Total	84	97	72.5	49	90	71.5	67	49	80.5	54.5	57	62	56.5	68.5
Procedural	Capture	87.5	100	83	42.5	100	66	53	41.5	na	33.5	17	na	37.5	60.1
	Restraint	50	89	50	39	89	66.5	41.5	75.5	89	75	100	100	na	72
	Procedures	82.5	89	91	45	96	75	76.5	84	94	90	57	67	79	78.9
	Recovery	100	100	100	50	100	75	75	75	100	75	100	100	100	88.5
	Ambience	50	80	80	50	30	50	40	47.5	52.5	77.5	55	100	67.5	60
	Scheduling	25	25	67	42	100	50	75	41.5	67	58.5	67	na	75	57.8
	Total	75	85.5	85	37	89	69.5	65.5	75.5	84.5	74.5	55	90	70.5	73.6
Culture of care	Initial training	90	84	97	85.5	90	74	80.5	84	91.5	77	96.5	94	81.5	86.6
	Continuing education	84.5	33.5	83.5	60	100	55.5	72.5	25	62.5	42	91.5	75	73.5	66.1
	Compassion fatigue and resiliency building programmes/activities	76.5	38	62.5	36	73	50.5	66.5	44	59	62	91.5	60	46.5	58.9
	Involvement/opportunity	71	38	74	69	81	51	50	46	68.5	52.5	79	67	50	61.3
	Choice and control in work schedule	75.5	88.5	78	58.5	72	57	54	51	73	67.5	84	83	64.5	69.7
	Recognition	85.5	69.5	79	67.5	75	76.5	80	75.5	83.5	79	96	94	84	80.4
	Voice concerns	86	95	83.5	76.5	83	75.5	71.5	70.5	72.5	97	91.5	92	62	81.3
	Competencies	82	91	84.5	81.5	64	69.5	54.5	80	88	70	100	96	82	80.2
	Bonus	21	21	7	21	0	63	91	70	21	35	0	35	14	
	Total	82	79	83	67	80	69	73.5	67.5	78.5	75.5	95.5	88	72	77.7
Total Score		82.5	89.5	87	63	87	76.5	78.5	64.5	78.5	70.5	85	84	71.5	

### Physical

The physical category aimed to evaluate physical and clinical health of primates as well as factors that may influence health. Overall sites scored the highest in the physical category compared to other welfare categories. Scores ranged from 72.5 to 98% (Table 3). The subcategory with the highest average score was general body condition (90.2%). The general condition descriptors where sites lost the most points were coat quality, animals reacting normally to external stimuli and personnel, animals having appropriate muscular development and fat deposits for sex and age, proper hydration, and signs of health conditions. The subcategory generally achieving the lowest score was records (81.7%), with lower scores seen for descriptors relating to the appropriateness of the bodyweight history of the animals based on their sex and age, and whether animal procedure history is readily available in the animal rooms. Feedback from personnel included that electronic records are generally available, thus records are not kept in the rooms but are readily accessible to employees. This suggested that the tool needed rewording to capture this possibility. For nutrition, lack of *ad libitum* feeding of base diet was the descriptor that most influenced scores for this category. Many of the sites record food intake as part of their study activities and thus provide a specific amount of diet for each animal, and all sites indicated that there are procedures in place to manage and observe animals with reduced appetite. For pain assessment and mitigation, the descriptor that was not met at all sites was “the facility has a specific policy or Standard Operating Procedure (SOP) concerning primate pain management practices based on current veterinary practices.” Feedback received on this descriptor was that some sites may have several policies or procedures for pain management and thus were not sure how to respond, or that the pain management policy was not specific to primates, but was generally applicable across species.

### Behavioural

The behavioural category aims to evaluate normal and abnormal primate behaviours and behaviour management inputs and sites scored from 60 to 97.5% in this category (Table 3). The subcategory with the highest score was social behaviour (85.2%), but within that subcategory, there were indicators that social group management can be challenging for sites, with some sites reporting evidence of fighting and bullying in social groups, and only six of 13 sites regularly assessed social groups and had established procedures to address incompatibility. The subcategory with the lowest average score across sites was ability to cope (74.9%), which has one descriptor that assessed “when separated from social partners for a procedure, primates do not perform abnormal behaviours.” For behavioural assessment, only seven of 13 sites indicated that personnel are specifically trained to identify normal and abnormal primate behaviours and that regular behavioural assessments are conducted. However, eleven of 13 sites had a team or individual specialised in primate behaviour. Sites reported the occurrence of abnormal behaviours in the animal behaviour category in 31.7% of the assessed rooms in Q1 2022 and 49.2% of the assessed rooms in Q3 2022. For feeding behaviour, there was only one descriptor: “primates are expressing natural feeding behaviours,” and only seven of 13 sites scored above 90% for this category, indicating some room for improvement in providing foraging opportunities. Only one site conducted breeding and had the ability to offer parental behaviour.

### Environmental

The environmental category aims to evaluate the quality of the space provided as well as resources and furnishings that permit primates to express species-typical behaviours and postures. Environmental was the lowest scoring category overall. One site scored 104% due to bonus points, while the rest ranged in score from 37 to 84.5% (Table 3). The three highest scoring sites were based in the UK and EU, and employ EU pen-style housing, rather than cage-style housing, which is more common in the US. The scores for the environmental category also revealed gaps in resources being provided to primates to provide comfort (e.g. thermoneutral or elevated resting surfaces) and encourage species-typical behaviours (e.g. foraging, social housing) and only a few sites provided regular out-of-cage exercise opportunities for animals.

### Training

The training category aims to assess procedures in place to prepare animals for study. The overall PWAT score for the training category ranged from 49 to 97% (Table 3). In general, acclimation periods are well established throughout facilities, with two sites indicating no procedure for environment acclimation upon arrival (because they are quarantine facilities) and four sites indicating that acclimation periods shorter than 14 days were permissible following animal arrival and depending on the source of the animals (e.g. local quarantine). There were a number of areas for improvement identified across sites in implementing more comprehensive behavioural management programmes. There were a number of descriptors within the subcategory habitution, desensitisation, and counter-conditioning for which sites scored below 2, such as habituation being conducted in a quiet environment and not paired with study activities, maintaining habituation over time, and documenting animal progress. In particular, the subcategory positive reinforcement training had an average score of 35.4%, indicating a need for more formalised training programmes to be implemented with primates to aid in co-operation and human-animal interactions. Additionally, in discussion with personnel, there were assumptions that positive reinforcement training referred specifically to clicker training. Clicker training is a method used in positive reinforcement training in which a device is used to mark the behaviour of interest with a click sound prior to providing the reinforcement. More clarification is needed on the different forms positive reinforcement can take in working with primates, such as simply providing a food reward for calm behaviour or for approaching personnel without the need for additional tools. For human interactions, a number of sites indicated that primates showed fear or stress towards personnel when staff are near the enclosure, emphasising a need to work on positive human-animal interactions.

### Procedural

The procedural category assesses refinements to research protocols. The overall PWAT score for procedure ranged between 37 to 90% (Table 3). The highest scoring subcategory was recovery (88.5%), which has one descriptor ‘following procedures, animals are monitored for pain indicators and to ensure animals return to normal state. Monitoring is performed by an individual familiar with the animals.’ The subcategories with the lowest score were capture (60.1%; ‘animals are comfortable and compliant with removal from home enclosure’) and ambience, which refers to noise levels and procedural space. In other categories there were a few descriptors for which sites consistently scored lower than 2, such as offering manipulanda during moderate restraint, providing rewards during or after procedures, training animals to take test articles voluntarily, minimising animal disturbance and personnel rushing by using strategic scheduling, and having sufficient space to allow animals and people to move without risk of injury, indicating issues that could be addressed across multiple sites.

### Culture of care

The culture of care category aims to assess employee satisfaction and training to prepare them for their responsibilities working with primates. The overall PWAT score for culture of care ranged from 67.5 to 95.5% (Table 3). The subcategory with the highest score was initial training (86.6%), which assessed the efficacy of the introductory training materials and learning environment. The subcategory with the lowest score was compassion fatigue and resiliency building programmes and activities (58.9%), highlighting an important gap for sites to enhance their compassion science programmes. Descriptors for which sites consistently scored below 2 were related to work-like balance, identifying programme leaders, and implementing internal programmes to honour the research animals.

Based on the results, a number of global trends were observed and general recommendations were created for how to address those gaps. These recommendations are provided in Table 4.

**Table 4.** General recommendations for improvement in each category based on the results of the 2022 Primate Welfare Assessment Tool benchmarking exercise

Physical	Behavioural	Environmental	Training	Procedural	Culture of care
-Monitor animal physical condition at a facility level over time to identify recurring issues and gaps in health monitoring programmes -Institute a subcommittee on pain management to review and update species-specific protocols	-Provide more primate-specific training on normal and abnormal behaviours -Be proactive in behavioural assessments to prevent abnormal behaviours from developing -Implement more social group management and compatibility assessments -Provide more employee training on affiliative and agonistic behaviours -Habituate primates to being separated from social partners during procedures -Encourage natural feeding behaviours, and provide improved opportunities for foraging and/or using other devices that encourage primates to work and explore to obtain food	-Adopt pen-style housing -Maximise vertical space for cage-style housing (i.e. balconies, tunnels) -Add additional furnishings such as visual barriers, high level perches, and swings -Add resources that promote comfort, natural behaviour, and stimulation (i.e. natural materials, thermoneutral surfaces, foraging opportunities, sensory and cognitive stimulation) -Provide opportunities for exercise outside of the home environment (especially for cage-style housing)	-Provide animals with 14-day acclimation period upon arrival before study activities begin -Develop formalised training protocols for habituating primates to study procedures using positive reinforcement training to encourage co-operation and decrease stress -Develop formalised process for monitoring animal progress and maintaining training throughout their time at the facility -Regularly evaluate training programmes for effectiveness -Personnel working with primates should be trained in use of operant conditioning techniques	-Implement habituation and positive reinforcement training protocols to improve animal comfort and compliance with restraint for procedures -Improve comfort and engagement for restraint devices -Refine techniques for repeat blood collections (e.g. use a catheter, peripheral vessels, microsampling, and/or monitor and rotate vein usage) -Minimise noise during procedures -Maximise space efficiency in procedural area -Avoid personnel rushing during procedures through thoughtful scheduling	-Provide more primate-specific educational opportunities during work hours -Provide more resources on compassion fatigue and resiliency building and advertise existing internal programmes -Involve personnel in primate resource evaluation and study refinement discussions -Allow personnel time for positive interactions with animals outside of study functions -Regularly evaluate employee recognition programmes to ensure personnel feel valued

## Discussion

The aim of this project was to develop a primate welfare assessment tool (PWAT) as a cross-facility collaborative effort to benchmark current primate management programmes and monitor forward animal welfare progress. Using a global focus group allowed the tool to be applied across facilities and helped build consensus and buy-in that aided in successful tool implementation. The tool was ultimately developed in a Smartsheet® platform, enabling semi-automated data management and visualisation. The PWAT incorporates input- and outcome-based welfare indicators as well as aspects of employee satisfaction, providing a holistic representation of primate management. The tool was used to benchmark primate programmes in 2022, and results were discussed and used by site-level stakeholders to develop goals to work towards and to ultimately create positive change for primate welfare.

Focus groups, as used in the development of the PWAT, have been used in a variety of fields such as education (Stathopoulou *et al.* 2019), health (Brouwers *et al.* 2019), and animal science (Ritter *et al.* 2021). Focus groups generally include participants recruited based on experience and expertise in a subject matter (Tausch & Menold 2016). Stakeholder focus groups are beneficial in driving animal welfare changes due to the complexity of animal welfare issues and the need to achieve buy-in from different levels of stakeholders to create lasting changes (Fernandes *et al.* 2019). To address complex welfare issues and create lasting change, there are five key areas to consider, including reflexivity of considering multiple perspectives, responsiveness of being able to adjust to changing expectations, revitalisation of reducing conflict by redirecting stakeholders to a common goal, resilience by maintaining flexibility, and relational capital by maintaining collaborations between stakeholders (Termeer *et al.* 2015; Fernandes *et al.* 2019). Utilising a stakeholder focus group in developing the PWAT addressed these key areas. The tool was developed to be flexible across sites, regions, and business purposes, and designed to require minimal training by making descriptors simple, self-explanatory, and well-detailed (CCAC 2019). Ongong discussions and collaborations occur through internal primate behaviour group listserves and quarterly meetings. The PWAT was created as a collaborative effort to ensure relevance and applicability in the research environment by individuals from various global sites, job titles, and experiences and built to be flexible and long-lasting to meet changing expectations.

The principle behind the PWAT is similar to the Extended Welfare Assessment Grid (EWAG) which uses a matrix to assesses animal welfare and cumulative suffering in research animals (Honess & Wolfensohn 2010). The EWAG uses similar welfare categories including clinical condition, behavioural deviations, environment, and experimental/clinical events. The main difference is that the EWAG focuses on assessing welfare at the individual level and monitoring cumulative suffering over time based on research use, whereas the PWAT is focused on assessing the overall primate behavioural management programme. Primates in research are used for more long-term studies compared to other research species, further emphasising the importance of a multifaceted approach to assessing welfare over time and considering cumulative suffering (Honess & Wolfensohn 2010; Paterson *et al.* 2023). Using a broader tool such as the PWAT could help identify gaps in the primate management programme that could result in animal-based indicators of poor welfare or cumulative suffering, while implementing a purely animal-based measure of welfare, such as the EWAG, could be used to monitor specific at-risk animals based on procedural severity and cumulative use, which could then be used to make decisions on humane endpoints (Honess & Wolfensohn 2010; Nunamaker *et al.* 2021).

The PWAT was developed through focus group discussions but alternative approaches exist. Recently, Truelove *et al.* (2020) identified 115 research macaque welfare indicators using a Delphi consultation process in which anonymous expert participants complete surveys over multiple rounds to achieve consensus on a topic. There are certain similarities between the Truelove and colleagues (2020) results and the PWAT. In Truelove *et al.* (2020), the welfare indices were split into six categories including enrichment, environment, health and management practices, appearance and health measures, behaviour, and physiology and genetics. In comparison to the PWAT, there are a similar number of indicators (PWAT = 133) and categories are comparable except that there was no integration of culture of care in the Delphi approach. For the Delphi approach, subject matter experts agreed that social enrichment and self-injurious behaviour were the most important indices of welfare (Truelove *et al.* 2020). Within the PWAT, there is a significant emphasis on social housing for which sites lose points for singly housing primates and indicators related to social housing are heavily weighted. In the Delphi method study, welfare indices were rated based on validity, reliability, and feasibility (Truelove *et al.* 2020). In the PWAT, the two main researchers (EAP, PVT) created the welfare descriptors and weighted them based upon welfare impact, then the focus group reached consensus by adding and removing some descriptors and adjusting the scoring weights. In the Delphi method study, it is suggested that assessing environmental-based measures (i.e. room temperature) is more feasible than animal-based measures (i.e. animal behaviour or health indicators) (Truelove *et al.* 2020). This is also true for the PWAT given that animal-based measures are direct measures of animal state and can be difficult to obtain, invasive, lengthy, and somewhat subjective (CCAC 2019). For example, quantifying behavioural abnormalities can be time-consuming and may differ between observers (Jirkof *et al.* 2020). However, in recent discussions on animal welfare assessment, there has also been greater focus on including individual animal assessments rather than just assessing at the group level to have holistic representation of overall animal welfare (Spangenberg & Keeling 2016; CCAC 2019; Winkler 2019), which was why it was deemed important to maintain animal-based measures at the room-level in the PWAT.

More recently, Prescott *et al.* (2022) took the 115 welfare indices identified by Truelove *et al.* (2020) and used a modified Delphi method to narrow the indices down to 56 and create a usable tool (GEN-MAC). The tool was used with a hypothetical scenario involving 500 primates but has not been tested in an animal facility (Prescott *et al.* 2022). In comparison to the PWAT, the GEN-MAC incorporates fewer and more simplified descriptors, which may make the tool faster to use. The scoring systems are similar, using scores of 0, 1, and 2. The GEN-MAC tool is currently available in Microsoft Excel®. For the PWAT, Smartsheet® was used to allow for the tool to be semi-automated such that personnel have immediate access to their raw data inputs, descriptive statistics, data visualisation, and historic data trends. These data are provided per category and subcategory, and at the site and company level allowing for a holistic and detailed view of the primate programmes in real time and historically.

The PWAT was specific enough to distinguish between facilities at a rather detailed level. For example, when comparing facility G and facility I in Table 2 (sites that have the same final score; 78.5%), one can see differences between facilities at the subcategory level in descriptors involving records, feeding behaviour, resources/enrichment provided, and restraint. This specificity allows for tailored recommendations to be made for each facility. Additionally, the PWAT was also able to identify global trends for more challenging aspects of research primate management as noted in Table 3, which will allow for development of global training resources to support site improvements. For example, for the ‘physical’ welfare category, to improve pain assessment and management, using tools such as the cynomolgus macaque grimace scale and specific behavioural indicators could be an important consideration for improving detection (Paterson & Turner 2022).

A unique component of the PWAT not found in most other welfare assessment tools is inclusion of a culture of care component. Creating a work environment in which employees feel valued and are satisfied with their jobs leads to better animal care and quality of science (Klein & Bayne 2007). In healthcare, and more recently, in veterinary medicine, culture of care in the work environment as well as compassion satisfaction and resilience, work-related satisfaction, and feeling valued have been recognised for their importance (Newsome *et al.* 2019; LaFollette *et al.* 2020; Randall *et al.* 2021). Areas that have been identified as beneficial to compassion satisfaction are work-life balance, positive interactions with research animals, involvement in animal-related refinements, continuing education opportunities provided during working hours, and feeling recognised and valued for their work (Randall *et al.* 2021; O’Malley *et al.* 2022). Using a formalised tool to evaluate and monitor the work environment for employees working with animals will benefit both animals and employees. In the PWAT, areas such as appropriate species-specific training and continuing education, access to compassion fatigue and resiliency building resources, the ability to be involved in animal welfare initiatives and voice concerns about animal welfare, work-life balance, and being recognised and feeling valued for their role in caring for animals and ensuring good animal welfare were all included to get a well-rounded assessment of the well-being of employees working with primates.

A limitation of the tool is that it was designed and tested in a contract research organisation environment in which cynomolgus macaques are the primary species worked with. It has not been tested for use with other primate species or in other environments with primates, such as in a zoo setting. Another limitation of the tool as it was designed and implemented is that while its use is required for sites working with primates, how the tool outcomes are used to change primate management programmes is not specified. Due to significant differences in facility function and operations (e.g. quarantine facility vs safety assessment facility) it is recognised that each facility will have unique strengths and areas for improvement, and that there will be asynchronous progress. The goal of the tool was not to diagnose issues and mandate changes, but to empower sites to evaluate their programmes, self-identify areas of improvement, and to prioritise and implement changes based on resource availability. As the sites assessed are all AAALAC accredited, welfare standards are already high. Therefore, the PWAT is meant to be an additional tool to encourage targeted refinement in primate management through discussions between various stakeholders and collaboration across facilities to share knowledge and experiences.

Research facilities are encouraged to incorporate welfare assessment tools and increasingly oversight bodies are requiring these as one means of ensuring continuous improvement in animal care and behavioural management programmes (CCAC 2021; Turner & Bayne 2023). As in the PWAT, assessments should include input- and outcome-based measures to ensure a holistic approach. Tools should be tested prior to implementation to ensure validity and feasibility and once the welfare assessment tool is implemented, the results should be shared with various stakeholders including animal care, behaviour, veterinary, and management personnel. Communication, discussion, and follow-up of the results among key stakeholders is important to promote ongoing refinements. The tool should be evaluated periodically with subject matter experts to ensure that the tool remains relevant and practical in assessing research primate programmes.

### Animal welfare implications

Welfare assessment is important for monitoring and improving animal care and use programmes within research environments. The tool includes input- and outcome-based measures for assessing research primate welfare, as well as measures of employee satisfaction and culture of care. The aim of the tool is to provide a means for holistic assessment of primate management programmes, which will allow facilities to identify gaps in their programmes to define and prioritise needed refinements. The tool was developed and launched in a global environment, and therefore has the potential to improve research primate welfare globally.

## Conclusion

Through an internal collaborative effort of primate experts, a primate welfare assessment tool (PWAT) was created and used at facilities that housed primates across a global business. The primate welfare assessment tool has six welfare categories that include physical, behavioural, environmental, procedural, training, and culture of care evaluations with a total of 133 welfare descriptors. The tool is composed of three forms: one that evaluates the behaviour management programme at the site level, one that evaluates individual animal states at the room level, and one that assesses the culture of care within a facility based on employee feedback. The PWAT successfully differentiated between programmes at different sites and identified areas for improvement at the facility and corporate level. Future directions for the PWAT will be to measure programmes over time, identify needed refinements, and overall improve research primate welfare.
